# Effect of a newly developed pastille on the salivary flow rate in subjects with dry mouth symptoms: a randomized, controlled, monocentric clinical study

**DOI:** 10.1186/s12903-021-01471-w

**Published:** 2021-03-12

**Authors:** S. Bielfeldt, D. Wilhelm, C. Neumeister, U. Schwantes, K. -P. Wilhelm

**Affiliations:** 1 proDERM Institute for Applied Dermatological Research, Kiebitzweg 2, 22869 Schenefeld, Hamburg, Germany; 2 Dr. Pfleger Arzneimittel GmbH, Dr.-Robert-Pfleger-Str. 12, 96052 Bamberg, Germany

**Keywords:** Medical device, Pastille, Dry mouth, Salivary flow rate, Saliva surface tension, Raman spectroscopy of saliva, Xerostomia

## Abstract

**Background:**

Xerostomia is associated with several diseases and is a side effect of certain drugs, resulting from reduced saliva secretion. Often, aged and sometimes younger people suffer from (idiopathic) xerostomia. Chewing gum and sucking pastilles may relieve symptoms of xerostomia by increasing the salivary flow rate due to the mechanical effect of sucking and gustatory stimulation. Swallowing problems and the urge to cough or experiencing a tickling sensation in the throat might be alleviated through a reduction in dry mouth symptoms. We investigated whether a pastille containing four polysaccharides increased the salivary flow rate and relieved the symptoms of dry mouth.

**Methods:**

Participating subjects with xerostomia were randomized into two equally balanced treatment groups. Subjects received the pastille on Day 1 and a control product (Parafilm®) on Day 3, or vice versa. Unstimulated saliva was collected every 2.5 min for 0–10 min. Stimulated saliva was collected after subjects sucked the pastille or the control product. The salivary flow rate was determined gravimetrically, and, in parallel, the feeling of dry mouth was assessed using a visual analog scale. Saliva surface tension was measured in pooled saliva samples (0–5 min of sampling). Additionally, in stimulated saliva from six subjects who sucked the pastille, the presence of the main ingredient—gum arabic—was examined by Raman spectroscopy.

**Results:**

Chewing the pastille significantly increased the mean salivary flow rate by 8.03 g/10 min compared to the mean changes after chewing the control product (+ 3.71 g/10 min; *p* < 0.0001). The mean score of dry mouth was significantly alleviated by the pastille (− 19.9 ± 17.9 mm) compared to the control product (− 3.3 ± 18.1 mm). No difference between the two products was seen regarding the saliva surface tension. Gum arabic was present in the saliva of all investigated subjects for up to 10 min after sucking the pastille.

**Conclusions:**

The pastille was well tolerated and effective in increasing the salivary flow rate and reducing mouth dryness after sucking. These results were in line with the detection of the main ingredient, gum arabic, in saliva for up to 10 min after sucking the pastille.

Trial registration German Register Clinical Trials (Deutsches Register Klinische Studien, DRKS) DRKS-ID: DRKS00017393, Registered 29 May 2019, https://www.drks.de/drks_web/navigate.do?navigationId=trial. HTML&TRIAL_ID = DRKS00017393.

## Background

Xerostomia is defined as dry mouth that results from a reduction in saliva secretion [1, 2]. Saliva plays an important role in oral health. It contains digestive enzymes, antimicrobial compounds, antibodies [3], electrolytes, buffering compounds [4] and mucoproteins in addition to being 99% water [5]. Saliva helps to prevent gingival mucosal erosions and ulcerations [6] and supports tooth remineralization [3]. Without normal salivary function, the risk of dental caries and other oral diseases (e.g., gingivitis, bad breath and bacterial overgrowth) is increased [6, 7]. Aged people often suffer from xerostomia. Xerostomia has also been related to several diseases [2], diabetes, alcoholic cirrhosis, cystic fibrosis, hormonal imbalance, autoimmune diseases, and disorders of the salivary gland. Additionally, the intake of several medications [1−3, 7], anticholinergics, diuretics, antihypertensives, anti-inflammatories, sedatives, anxiolytics, antihistamines, opioid analgesic agents, and radiation therapy to the head and neck may lead to xerostomia [8]. Social and psychological factors (e.g., anxiety, stress, and depression) are also possible causes of the feeling of a dry mouth. Patients suffering from xerostomia often complain about a burning mouth, taste disturbances and denture discomfort [2]. Dry mouth may have an impact on a person’s quality of life, especially when it affects speech, leading to a loss of taste or sleep disturbances [9–11].

The salivary flow rate is an important marker for xerostomia. The average unstimulated whole salivary flow rate in healthy subjects ranges from 0.3 to 0.5 mL per minute [12]. Flow rates of 0.1 mL per minute or less are considered to indicate hyposalivation or xerostomia [13].

Sucking of a pastille or chewing a piece of chewing gum may increase the salivary flow rate in two ways: via mechanical stimulation by sucking of the product and by a gustatory effect due to the release of flavors. Thus, the mouth becomes less dry, and the urge to cough, a tickly throat or swallowing problems, might be alleviated due to the viscous and adhering solution.

Unstimulated natural saliva has low surface tension [14]. Low salivary surface tension is beneficial because it leads to full coating of the oral cavity and results in tight and strong interactions between the saliva and the oral mucosa [4]. Moreover, a decrease in saliva surface tension may be valuable in patients with caries, as shown by Kazakov et al. [15], who found increased surface tension of saliva in children with caries compared to children without caries.

Saliva stimulated by mechanical sucking or chewing alone has different rheological properties and does not generate the same mucoadhesive film as unstimulated saliva [4, 16]. In xerostomia, the content of the gel forming polysaccharide hyaluronic acid in saliva is decreased [17]. Effective treatment of xerostomia, therefore, includes not only the stimulation of saliva but also an improvement of its composition. Polysaccharides such as carboxymethyl cellulose and hyaluronic acid are well known for improving mucoadhesion [18–20].

The investigated pastille contains a combination of polysaccharides (Table 1), which are supposed to create a moisturizing and protective film on the oral mucosa when it is sucked and dissolved [14]. As the pastille achieves its intended action primarily by a physical mode of action without pharmacological effects, it is categorized as a medical device according to the definition of the European Union and the United States. It is indicated for the relief of mouth dryness, hoarseness, tickling of the throat, other painful symptoms in the mouth or throat and swallowing difficulties. Side effects of the respective pastilles are rare; in sensitive subjects, side effects may occur due to hypersensitivity reactions to ingredients in the product [21].

**Table 1:** Investigational products

Pastille (medical device)	ipalat Hydro Med
Ingredients	Active ingredients: gum arabic, natrosol, sodium hyaluronate, and carrageenan
	Further ingredients: maltitol syrup, honey flavor, primulae extract, bitter fennel oil, star anis oil, and sucralose
Administration	1 pastille per subject, sucking and chewing
Manufacturer	Dr. Pfleger Arzneimittel GmbH D-96045 Bamberg, Germany

Control	Parafilm "M"
Ingredients	Paraffin wax and polyolefins
Administration	5 × 5 cm piece per subject, sucking and chewing
Manufacturer	Bemis Corporate, 2301 Industrial Drive, Neenah, WI 54956, USA

In this randomized controlled clinical study, we investigated the stimulating effect of a pastille on the salivary flow rate and assessed its effects on the subjective feeling of dry mouth. Additionally, we assessed the influence of a pastille on the surface tension of the saliva. To correlate the observed effects with the presence of the main ingredient—gum arabic—this component was detected in the saliva samples at several time points after sucking.

## Methods

This monocentric, randomized, controlled, crossover study was carried out at the proDERM Institute for Applied Dermatological Research in June 2019. For reporting of the study results, we adhered to the CONSORT guidelines.

### Investigational products

The investigational products are described in Table 1. The control product, Parafilm "M", is a tasteless wax that has been used in other studies for mechanical stimulation of saliva [4].

### Randomization and blinding

Subjects admitted to the trial were randomized into two equally balanced treatment groups. Additionally, the timely assignment of the test products was equally balanced. According to the randomization plan, the subjects received the control product on Day 1 and the pastille on Day 3, or vice versa. Here, randomization to the two groups served only to ensure that the transition between the two treatments was evenly distributed. A comparison between the groups did not take place as the study was only based on a crossover design. The method of randomly permuted blocks of fixed size ensured that equal, or almost equal, numbers of subjects were assigned to each treatment group. Randomization codes were generated centrally by proDERM GmbH utilizing the software SAS® for Windows (version 9.4). Upon enrollment, each subject was given a unique number corresponding to the randomization code. The randomization specified the allocation of the control product and the pastille to each subject on Day 1 and Day 3.

Due to the nature of the pastille (taste and appearance), the study could not be performed blindly. However, since the person who carried out the measurements was not aware of which product was used, this individual was blinded. The data manager, principal investigator and trial statistician also remained blinded until all queries were resolved and the database was closed.

### Subjects

Twenty-six Caucasian subjects (19 females, 7 males; median age 52.5 years, range: 27–74 years) with xerostomia (otherwise healthy oral cavity, including the tongue and gingival mucosa) participated in this trial. If females were of childbearing age, they were only included in the study after providing a negative pregnancy test [urine test for human chorionic gonadotropin (hCG)] at the time of admission. Subjects with generally poor health conditions were excluded. Analysis of the main ingredient, gum arabic, in saliva by means of Raman spectroscopy was carried out to investigate saliva samples of a subgroup of 6 subjects who sucked the pastille.

The main inclusion criterion of having a dry mouth was evaluated by subjective grading using a visual analog scale (total length 100 mm) ranging from "not dry" to "extremely dry". Eligible subjects had to have a dryness score of at least 40 mm. The reason for dry mouth was documented if known. Participants named smoking, menopause, climate conditions, dehydration due to low fluid intake, medication, diabetes, mouth breathing, and wearing dentures as reasons for dry mouth. Further inclusion criteria were providing written informed consent, avoiding heavy sweating, not eating spicy or salty foods one day prior to the measurements, not consuming alcohol extensively in the evening prior to the measurements, avoiding inner excitement in the morning prior to the measurements, avoiding food with a saliva-reducing or saliva-stimulating effect, avoiding food requiring extensive chewing, not smoking 4 h prior to the measurements, not changing one’s prosthesis or artificial dentition throughout the course of the study, agreeing to inform the study site in case of changes in therapies especially regarding medication which can cause xerostomia, and having a negative urine pregnancy test.

The main exclusion criteria were as follows (in addition to general exclusion criteria in clinical trials):

suffering from diseases of the salivary glands, dental or other problems within the oral cavity; exhibiting chewing or swallowing problems; and intaking medications and food or participating in activities that had an impact on the salivary flow or having an active disease at the test area (e.g., tumors).

Smoking was prohibited within the 4 h before the scheduled visits as was the intake of any food or beverages (except water) within 90 min prior to the measurements.

### Assessment of dry mouth

Subjects were asked to evaluate the sensation of dry mouth on a visual analog scale (VAS) for inclusion in the study, on Day 1 and on Day 3 before and after sucking the investigational products at the study site.

### Salivary flow rate measurement

On study Day 1 and Day 3, the salivary flow rate was determined twice. Initially, the unstimulated salivary flow rate was assessed (baseline). After a short rest period of at least 5 min, the subjects either sucked the pastille or the control product, and subsequently, the stimulated flow rate was assessed. The collection of saliva was performed according to the noninvasive "spitting method" of Navazesh and Kumar [22] as described briefly below.

To assess the unstimulated salivary flow, the subjects were asked to spit into plastic tubes at 30 s intervals. Saliva was collected in separate tubes after 2.5, 5, 7.5 and 10 min. After a short break, the stimulated salivary flow rate was assessed. Therefore, the assigned treatment, pastille or control product, was sucked until it was dissolved (no longer than 5 min), or the control product was chewed up to a maximum time of 5 min. The subjects again spat at 30 s intervals into tubes. Saliva was collected in separate tubes after 2.5, 5, 7.5 and 10 min starting at the beginning of sucking.

At the next visit, the procedure was repeated with the second assigned product.

The weight of each saliva sample was determined, and the salivary flow rate (unstimulated and stimulated) was calculated as the sum of all collected saliva samples per measurement time (2.5, 5, 7.5 and 10 min). The primary variable was the total salivary flow rate determined after 10 min (weight in grams over 10 min).

The weight of each saliva sample was assessed using an analytical balance (SCALTEC SBC31, SCALTEC Instruments GmbH, Heiligenstadt, Germany).

The primary endpoint was the difference between treatment groups in the change in the salivary flow rate (grams per 10 min) from unstimulated to stimulated conditions.

### Salivary surface tension measurement

Salivary surface tension is a measure of the ability of saliva to spread into the oral cavity and consequently to form a protective biofilm on the mucous membrane (mucoadhesion). A low value indicates the presence of broad and strong interactions between the oral mucosa and the biological fluid, which allows saliva to fully coat the oral cavity. Salivary surface tension was measured with the SITA Science line t100 (SITA Messtechnik GmbH, Dresden, Germany) by use of the bubble pressure method.

Due to cohesive forces within a liquid, air bubbles within the liquid are compressed, and this pressure depends on the surface tension of the liquid. Bubbles are produced by the instrument by pumping air across a capillary that is immersed in the saliva sample. The bubble pressure first rises to a maximum (hemisphere size of the bubble) and then returns to the initial pressure when the bubble forms a sphere and is released from the capillary tip. The pressure difference (maximum pressure minus initial pressure) is recorded. The pressure difference is linearly correlated with surface tension. As a secondary parameter, the surface tension of stimulated saliva was measured in the pooled saliva samples that were obtained after 5 min of sampling. On Day 1 and Day 3, salivary surface tension was measured in samples already weighed to assess the salivary flow rate. The sampling volume had to be at least 3 ml to enable correct measurements of salivary surface tension. In the case that the collected saliva volume for each of the samples (pastille or control) after 5 min of sampling was less than 3 ml, the respective subject had to be excluded from this analysis. Since it was expected that a sampling volume of 3 ml of unstimulated saliva could not be collected in 5 min, only stimulated saliva samples were investigated.

### Detection of gum arabic in saliva samples by Raman spectroscopy

The major component of the pastille, gum arabic, was determined ex vivo in the saliva directly after sampling in the clinical setting. To avoid precipitation in the saliva samples or other complications due to storage times that were too long, measurements had to be performed as quickly as possible. Only small samples could be taken, as most of the saliva samples had to be saved for the surface tension measurement; therefore, highly sensitive confocal Raman spectroscopy [23, 24] was used.

On Day 1, detection of gum arabic was performed in saliva samples of a subgroup of the first 6 subjects. Therefore, a small amount (50 µl) of the harvested saliva at baseline, 5, 7.5 and 10 min, which had already been weighed to assess the salivary flow rate, was retrieved.

A small droplet of the saliva (approx. 10 µl) was applied to the glass plate of the RiverD Raman spectrometer (RAMAN SPECTROMETER gene2-SCA Ultimate, River Diagnostics, Rotterdam, Netherlands) to qualitatively assess the amount of gum arabic in the sample. Raman spectra were obtained by focusing the low-power laser beam 30 µm into the small droplet of saliva and by measuring the Raman scattered light at the laser focus. The Raman spectrum of the fingerprint region (400–1800 cm^−1^) was recorded. The quantitative major component of the pastille, gum arabic, has a unique well-known Raman spectrum [25, 26]. The resulting spectrum was printed and visually analyzed for the presence of gum arabic by comparing the measured spectra to spectra of saliva with known concentrations of the dissolved pastille.

### Assessment of tolerability

On Day 1 and at the final visit (Day 3), oral tolerability of the investigational products being investigated was assessed by the investigator regarding erythema, edema/infiltration and erosion (each parameter separately) on a 4-point scale (0 = absent, 1 = mild, 2 = moderate, and 3 = severe).

Additionally, the subjective tolerability (tension sensation, burning, and tickling sensation) was rated by the subjects on a 6-point scale (0 = none, 1 = mild and intermittent, 2 = moderate or continuous, 3 = severe (independent of duration), 4 = intermittently insufferable (> 5 min), 5 = continuously insufferable) after each product was used.

### Assessment of safety

#### Physical examination(s) focused on the oral cavity

The tongue (fissures), oral cavity and gingival mucosa (erythema, gloss, and others) were examined by a trained technician or physician at the screening and final visit (Day 3). The examination was performed and the result was either "yes" or "no" regarding whether good health was indicated. In the case of "no", further explanation was needed.

#### Blood pressure, pulse rate and pregnancy test

Blood pressure and pulse rate were determined at screening and at the last visit. These variables were assessed in the resting and seated subjects using a semiautomatic measurement system (Boso "medicus"; Bosch & Sohn, Juningen, Germany).

### Study schedule

The study schedule including all procedures performed on the study days is listed in Table 2.

**Table 2:** Study schedule and procedures

	Screening Day − 7 to − 1	Day 1	Day 3
Informed consent	X		
Demographical data, medical history and pregnancy test	X		
Medical history of dry mouth	X		
Physical examination of the oral cavity	X		X
Vital signs (blood pressure and pulse rate)	X		X
Concomitant diseases and medication/treatment	X		
Inclusion and exclusion criteria	X	X	
VAS assessment of dry mouth	X	X^1)^	X^1)^
*Saliva sampling for salivary flow measurement*		X	X
(1) Unstimulated (baseline measurements)			
(2) Rest period (at least 5 min)			
(3) Stimulated			
Salivary surface tension measurement		X	X
Raman spectroscopy (subgroup, n = 6)		X	
Tolerability assessment (locally by an investigator and subjective by the subject)		X	X
Adverse events and change in concomitant medication		X	X

Before and after sucking

### Statistical analysis

#### Primary endpoint and estimation of sample size

A review of existing data suggested that a mean salivary flow rate of 6 g/15 min with a standard deviation of 5.5 seemed reasonable for the group treated with the pastille. Furthermore, a mean salivary flow rate of 3 (g/15 min) with a standard deviation of 2.5 was assumed for the control group. Under the assumption that the difference between the control and pastille groups at the 10-min assessment time point would be similar to that at 15 min, the following hypothesis was tested with a paired t-test: H0: µP = µC versus H1: µP ≠ µC, where µP denotes the mean difference from baseline of the salivary flow rate (g/10 min) for the pastille, and µC denotes the mean difference from baseline of the salivary flow rate (g/10 min) for the control group. To detect a difference in the salivary flow rate (g/10 min), a sample size of at least 22 subjects was required to obtain a power of at least 80% given a significance level of 5%. To take into account a dropout rate of 20%, a total of 26 subjects were included in the study. Primary analysis was based on the per protocol population (PP). The same analysis was repeated for the full analysis set (FAS) to assess the consistency of the results.

#### Secondary endpoints

For the secondary analysis, only the values measured from the pastille group could be used, since in the control group, not enough saliva was obtained to make a valid measurement. Due to this issue, the secondary endpoints were evaluated using descriptive statistical methods. Accordingly, the results were interpreted in an exploratory context.

A further peculiarity arose for the analysis of the Raman spectroscopy data. For this analysis, a subgroup of 6 subjects was analyzed after these individuals had sucked the pastilles. Here, too, a descriptive statistical methodology was used for analysis, and the classification of the results was also carried out from an explorative point of view.

## Results

### Disposition of subjects

Out of 35 screened subjects, 9 did not meet the inclusion/exclusion criteria. All 26 enrolled subjects completed the study as valid cases without major protocol violation.

### Salivary flow rate

The main objective of the study was to investigate the change in salivary flow rate after 10 min of sucking/chewing the pastille and the control product.

After sucking/chewing the pastille, the mean salivary flow rate increased by 8.03 [g/10 min] from 2.03 g/10 min when unstimulated to 10.06 g/10 min, whereas after sucking/chewing the control product, the salivary mean flow rate increased by 3.71 g/10 min, from 2.13 g/10 min when unstimulated to 5.85 g/10 min (Table 3).

**Table 3:** Salivary flow rate (g/10 min)

Salivary flow rate [g/10 min], means, standard deviations and device comparisons on differences from baseline (unstimulated) by paired t-test					
Analysis set (FAS)			Mean values ± SD		*p* values
Phase	Device	n	Raw data	Differences from baseline (unstimulated)	Comparison of the pastille vs. the control product
Unstimulated	Pastille	26	2.03 ± 1.40	–	–
	Control	26	2.13 ± 1.36	–	
Stimulated	Pastille	26	10.06 ± 4.14	8.03 ± 3.66	< 0.0001
	Control	26	5.85 ± 3.27	3.71 ± 2.45	

FAS = full analysis set, SD = standard deviation

Bold *p* value: significant (*p* ≤ 0.05)

**Fig. 1:**
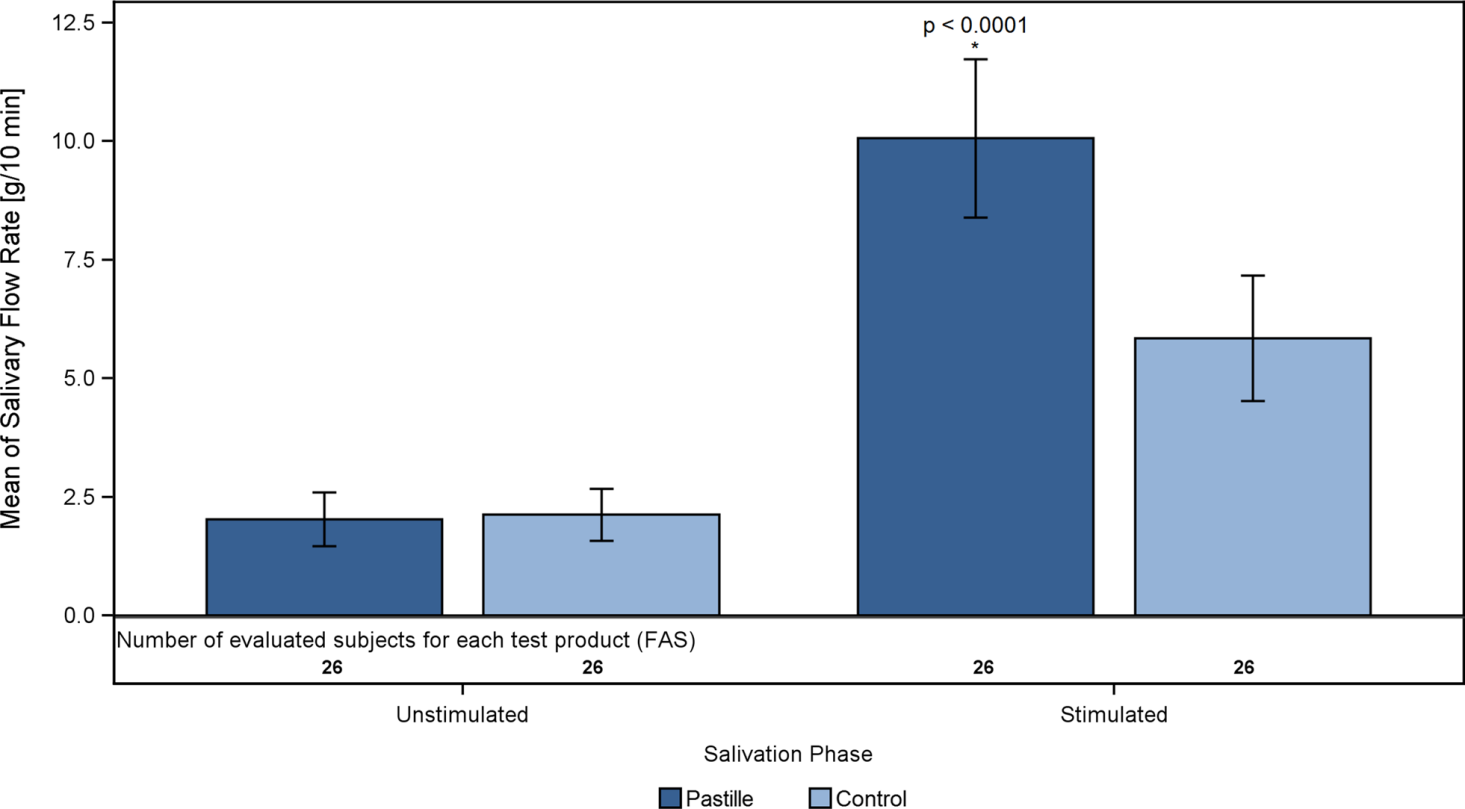
Unstimulated and stimulated salivary flow rates (mean values and SD) of the pastille (dark blue) and control product (light blue) groups

In Fig. 1, the observed mean flow rates, standard deviations and test statistics are displayed. After sucking the pastille/control product for 10 min, a significant increase in the salivary flow rate in the pastille group compared to the control group was observed (paired t-test; *p* < 0.0001).

### Salivary surface tension

As a secondary parameter, salivary surface tension was measured in the pooled stimulated saliva samples that were obtained after 5 min of sampling. Data from 25 subjects could be evaluated. The salivary surface tension obtained after sucking the pastille was measured and had an average of 65.80 mN/m with a standard deviation of 17.81 mN/m, whereas for the control product, values of 62.04 mN/m with a standard deviation of 9.00 mN/m were determined. The Wilcoxon signed-rank test revealed no statistically significant difference (*p* = 0.5350).

### Assessment of dry mouth

The subjective assessment of dry mouth on a VAS scale was assessed as a secondary parameter (Table 4). At the time of inclusion, all subjects suffered from a feeling of dry mouth of ≥ 40 mm [VAS]. In the pastille group, the mean score decreased by 19.9 mm (standard deviation 17.9 mm) from a mean score of 69.0 mm before sucking to 49.2 mm after sucking, whereas for the control group, a mean decrease of 3.3 mm (standard deviation 18.1 mm) was found (from 71.2 to 67.8 mm).

**Table 4:** Subjective assessment of the feeling of dry mouth [VAS]*

Subjective assessment of the feeling of dry mouth [VAS], means, standard deviations and device comparisons on differences from baseline (before sucking process) by paired t-test					
Analysis set (FAS)			Mean values ± SD		*p* values
Phase	Device	n	Raw Data	Differences from baseline (before the sucking process)	Comparison of the pastille versus the control product
Before the sucking process	Pastille	26	69.0 ± 18.4	–	–
	Control	26	71.2 ± 14.7	–	
After the sucking process	Pastille	26	49.2 ± 22.9	− 19.9 ± 17.9	0.0016
	Control	26	67.8 ± 18.2	− 3.3 ± 18.1	

*At the time of inclusion, all subjects suffered from a feeling of dry mouth ≥ 40 mm [VAS]

Bold *p* value: significant (*p* ≤ 0.05)

Statistical analysis confirmed normally distributed data for the score differences. The paired t-test revealed a significant difference between the two treatments (*p* = 0.0016), confirming the superiority of the pastille.

### Assessment of tolerability

Local tolerability was evaluated by assessing erythema, edema and erosions in the oral cavity. One case of erythema and one case of erosion were observed after sucking the pastille, whereas one case of erythema and two cases of erosion were seen after chewing the control product. Only one out of these four subjects reported mild erosion on both days (Day 1: control product and Day 3: pastille). As the oral cavity was examined prior to administration of the investigational product, a carry-over effect could be excluded.

Subjective tolerability was assessed by the subjects themselves, evaluating the parameters tension sensation, burning and tickling sensation. Two mild and two moderate reactions were reported after sucking the pastille, whereas five mild reactions and one moderate reaction were present after chewing the control product.

### Detection of gum arabic in saliva samples

The presence of the quantitative major ingredient, gum arabic, in the saliva samples of a subgroup of 6 subjects was determined by Raman spectroscopy after they sucked the pastille; samples were taken at baseline, 5, 7.5 and 10 min after sucking. Five and 7.5 min after sucking, gum arabic was detected in all 6 saliva samples by Raman spectroscopy, and it was still present in two samples after 10 min of sucking. In Table 5, the number of saliva samples with positive detection of gum arabic is given as a percentage at each assessment time point.

**Table 5:** Detection of gum arabic in saliva samples by Raman spectroscopy

Analysis set (FAS)	Presence of gum arabic in saliva assessed by Raman spectroscopy (N = 6)					
	Counts and percentages					
	Yes		No		Total	
	n	(%)	n	(%)	n	(%)
At baseline	1	16.7	5	83.3	6	100.0
After 5 min	6	100.0	0	0	6	100.0
After 7.5 min	6	100.0	0	0	6	100.0
After 10 min	2	33.3	4	66.7	6	100.0

FAS = full analysis set

In one saliva sample, a weak spectrum for gum arabic was identified at baseline. Possible reasons may be that the subject chewed/ate something containing gum arabic (not adhering to the inclusion/exclusion criteria).

## Discussion

In this study, a pastille (ipalat® Hydro Med) was investigated for its effects on saliva secretion, changes in salivary surface tension, relief of dry mouth symptoms, and tolerability in comparison to a control product (parafilm "M"). Additionally, the presence of its main ingredient, gum arabic, was detected.

Twenty-six subjects with symptomatic dry mouth were enrolled. In the study population, the basal unstimulated salivary flow rate of 0.203 g/min in the pastille group, 0.213 g/min in the control group, and a range of 0.3 to 0.5 g/min in the normal population supports the subjective assessment of dry mouth [12].

The subjects sucked or chewed the pastille as well as the control product for no longer than 5 min. After sucking the pastille, the salivary flow rate increased significantly by 8.03 g/10 min, whereas the increase in salivary flow rate after chewing the control product for the same time was much less (increased by 3.72 g/10 min, N.S.). Therefore, the primary goal of the study was achieved. The increase in salivary flow rate after chewing the control product indicates that a mechanical stimulus alone is sufficient for a slight increase in salivary flow [27, 28]. However, there must be an additional effect of the pastille to obtain a significant (*p* < 0.0001) increase in the salivary flow rate. Most likely, the release of the flavors of the pastille has an additional gustatory stimulating effect on the salivary flow rate. Previously, it was described that after chewing different types of chewing gum, an initial increase in the flow rate is probably induced by gustatory flavors. A decline in flow rate has been shown to be related to the loss of taste [29]. However, others have shown that with chewing gum, the main effect on the flow rate seems to be caused by chewing/sucking through stimulation of periodontal mechanoreceptors [27].

The assumption that an increase in the salivary flow rate would alleviate the feeling of dry mouth was supported by the subjective assessment of the subjects themselves. The feeling of dry mouth decreased significantly after sucking the pastille compared to chewing the control product (*p* = 0.0016). Symptoms of dry mouth might have been alleviated due to the moisturizing, less viscous and more adhesive solution present after sucking the pastille. The entire composition of the pastille, gum arabic, hyaluronic acid, natrosol and carrageenan, seems to be important for this effect.

A low surface tension of saliva is desired to improve wetting of the oral mucosa in patients with dry mouth. Our preliminary investigations showed that dissolving the pastille in artificial saliva reduced the surface tension of the artificial medium by 6 mN/m compared to the results obtained using purified water. Mystkowska et al. [3] showed that artificial saliva solutions containing phosphate buffered saline and porcine mucine and/or poloxamer and/or the polysaccharide guar gum can have a surface tension close to or significantly different from natural saliva depending on the method of buffer preparation and the additional ingredients. We examined whether there was an impact of the polysaccharides in the pastille on the surface tension of saliva in vivo. We found no relevant influence of the pastille on the rheological parameter. Our rheological values after sucking the pastille (65.80 mN/m ± 17.81 mN/m) were comparable to those of the control product (62.04 mN/m ± 9.00 mN/m). These results are also in line with those of Gittings et al. [4], who also did not find significant differences in the surface tension of unstimulated and stimulated saliva by chewing Parafilm; however, the authors detected differences in other parameters, such as viscosity and pH. Consequently, surface tension does not seem to be a crucial parameter in saliva rheology.

The Raman spectrum of gum arabic, which is the main ingredient of the pastille used in this study, is specific, well documented in the literature and provides a quick and precise method of detecting gum arabic in saliva or other body fluids.

Five and 7.5 min after sucking the pastille, gum arabic was detected in all saliva samples, whereas 10 min after sucking, it was still present in two saliva samples. This means that gum arabic and the combination of colloid ingredients provided a long-lasting beneficial impact on the moisturizing film and on the salivary flow when the pastille was sucked.

The pastille was well tolerated based on the investigator’s and the subjects’ tolerability assessment. Two subjects experienced mild reactions in the pastille group and two in the control group. Therefore, we assume that the pastille is suitable for use in xerostomia cases.

## Conclusion

The pastille significantly increased the salivary flow rate at all sampling time points compared to the salivary flow rate observed when subjects chewed the control product. Moistening of the oral cavity was achieved, and the feeling of dry mouth was significantly improved. The major ingredient of the pastille, gum arabic, was present in the saliva for up to 10 min after sucking. The pastille was well tolerated in this study. We conclude that the pastille is very suitable for alleviating xerostomia.

## Data Availability

The datasets used and/or analyzed during the current study as well as the full trial protocol are available on reasonable request from Dr. Pfleger Arzneimittel GmbH.

## Abbreviations

cm: Centimeter
CMC: Carboxymethyl cellulose
CRF: Case report form
CRO: Contract Research Organization
DRKS: Deutsches Register Klinische Studien (German Register for Clinical Studies)
FAS: Full analysis set
g: Gram
GCP: Good clinical practice
H0: Null hypothesis
H1: Alternative hypothesis
HA: Hyaluronic acid
hCG: Human chorionic gonadotrophin
IEC: Independent Ethics Committee
ISO: International Organization for Standardization
m: Meter
Max: Maximum
MEDDEV: European Medical Device Vigilance System
Min: Minimum
µl: Microliter
mL: Milliliter
mm: Millimeter
mN: Millinewton
proDERM ID: ProDERM identification
PP: Per protocol
SD: Standard deviation
SOP: Standard operating procedure
SP: Safety population
VAS: Visual analog scale
